# Human umbilical cord mesenchymal stem cells promoting knee joint chondrogenesis for the treatment of knee osteoarthritis: a systematic review

**DOI:** 10.1186/s13018-023-04131-7

**Published:** 2023-08-29

**Authors:** Pengwei Zhang, Bo Dong, Puwei Yuan, Xun Li

**Affiliations:** Xi’an Hong Hui Hospital, Xi’an, Shaanxi China

**Keywords:** Osteoarthritis, Knee, Chondrocytes, Regenerative medicine, Exosomes, Safety, Inflammation

## Abstract

**Purpose:**

The onset of OA is affected by a variety of factors, which eventually lead to the loss of cartilage in the joints, the formation of osteophytes, the loss of normal knee mobility, and pain and discomfort, which seriously affects the quality of life. HUC-MSCs can promote cartilage production and have been widely used in research in the past decade. This article systematically summarizes that it is well used in basic research and clinical studies to promote inflammatory chondrogenesis in the treatment of OA. Provide a theoretical basis for clinical treatment.

**Patients and methods:**

This study collected CNKI, Wanfang, PubMed, and articles related to the treatment of OA with HUC-MSCs since their publication, excluding non-basic and clinical studies such as reviews and meta-analysis. A total of 31 basic experimental studies and 12 clinical studies were included. Systematically analyze the effects of HUC-MSCs on inhibiting inflammatory factors, promoting chondrocyte production, and current clinical treatment.

**Results:**

HUC-MSCs can reduce inflammatory factors such as MMP-13, ADAMTS-5, IL-1β, IL-1, IL-6, TNF-α, induced conversion from M1 to M2 in OA to protect cartilage damage and reduce OA inflammation. Synthesize ColII, SOX9, and aggrecan at the same time to promote cartilage synthesis.

**Conclusion:**

HUC-MSCs not only have typical stem cell biological characteristics, but also have rich sources and convenient material extraction. Compared with stem cells from other sources, HUC-MSCs have stronger proliferation, differentiation, and immune regulation abilities. Furthermore, there are no ethical issues associated with their use. Safety: Primarily attributed to pain, the majority of individuals experience recovery within 24 h following injection. HUC-MSCs possess the ability to alleviate pain, enhance knee joint function, and potentially postpone the need for surgical intervention in both non-surgical and other cases, making them highly deserving of clinical promotion and application.

## Introduction

Knee osteoarthritis (OA) is a chronic disease in which aging or external stimulation leads to degenerative changes of the knee joint [1]. The early clinical manifestations are joint swelling, pain, and limited movement. In the late stage, subchondral hyperplasia occurs, osteophytes form, the knee joint space narrows, and the functional structure of the knee joint undergoes changes. The main pathological manifestations were cartilage injury of knee joint, synovial hyperplasia, and abnormal hyperplasia of subchondral bone. Articular cartilage injury is most common in patients with OA. The peculiarities of the articular cartilage structure determine its poor self-repair after injury. Therefore, the treatment of OA cartilage injury is a wide range of clinical difficulties, and also the main cause of restricted knee activity in the elderly [2]. It is estimated that at least 10% of people over the age of 60 worldwide have OA. As the world’s population ages, OA patients will continue to increase [1]. The global prevalence of OA among people aged 50 years and older is 14%-38% in women and 4–14% in men. The prevalence of the disease is expected to rise due to increased risk factors such as obesity and advanced age. OA has been linked to genetic factors, mechanical stress, and chondrocyte differentiation. While it is predominantly seen in individuals aged 65 and older, there has been a noticeable increase in its prevalence among those under 65 in recent times [3]. Numerous studies have provided evidence that intra-articular injections of various substances, such as sodium hyaluronate, glucocorticoids, platelet-rich plasma, and stem cells, can effectively decrease systemic side effects and provide more targeted and direct effects by delivering drugs directly into the affected joint. Compared to oral NSAIDs and other systemic pharmacological treatments, local injection therapy has been shown to be more effective [4].

With the continuous discovery of novel biological cells, nanomolecules, cell molecules, and biomaterials, an increasing number of innovative cellular types are being progressively utilized in various branches of medicine [5]. For instance, human umbilical cord mesenchymal stem cells (HUC-MSCs) have found application in diverse medical domains [6]. HUC-MSCs not only have typical stem cell biological characteristics, but also have rich sources and convenient material extraction. Compared with stem cells from other sources, HUC-MSCs have stronger proliferation, differentiation, and immune regulation abilities, and there are no ethical issues [7]. HUC-MSCs are obtained through subculturing neonatal umbilical cord specimens, typically involving the propagation of stem cells over 3–5 generations [8]. HUC-MSCs have been widely used in the treatment of OA in the past decade of research [9]. And it has achieved good results in promoting articular cartilage in cell experiments, animal experiments, and clinical studies [10]. HUC-MSCs can not only differentiate into chondrocytes to promote the generation of joint cartilage, but also secrete a large number of cytokines to participate in the inflammatory immune regulation process, making HUC-MSCs an ideal seed cell for treating OA cartilage injury [11].

## Material and methods

This study uses “Osteoarthritis” and “Mesenchymal Stem Cells” as the main search terms, Using “Osteoarthritis,” “Osteoarthritis,” “Osteoarthrises,” “arthritis generative,” “Degenerative Arthritis,” “Degenerative Arthritis,” “Arthrosis,” “Arthroses,” “Osteoarthris Deformans” and “stem cell mesenchymal,” “Mesenchymal Stem Cell,” “Bone Marrow Mesenchymal Stem Cells,” “Bone Marrow Mesenchymal Stem Cells’ Cell,” “Bone Marrow Stromal Cells,” “Bone Marrow Stromal Cell.” The secondary search terms include “bone marrow stromal cells multipotent,” “Multipotent Bone Marrow Stromal Cell,” “Multipotent Bone Marrow Stromal Cells,” “advertisement derived mesenchymal stem cells,” “advertisement derived mesenchymal stem cells,” “advertisement derived mesenchymal stromal cells,” and “advertisement derived mesenchymal stromal cells.” Searches were conducted on PubMed, CNKI, Wanfang, and other databases.

## Results

### Selection of articles

**Fig. 1:**
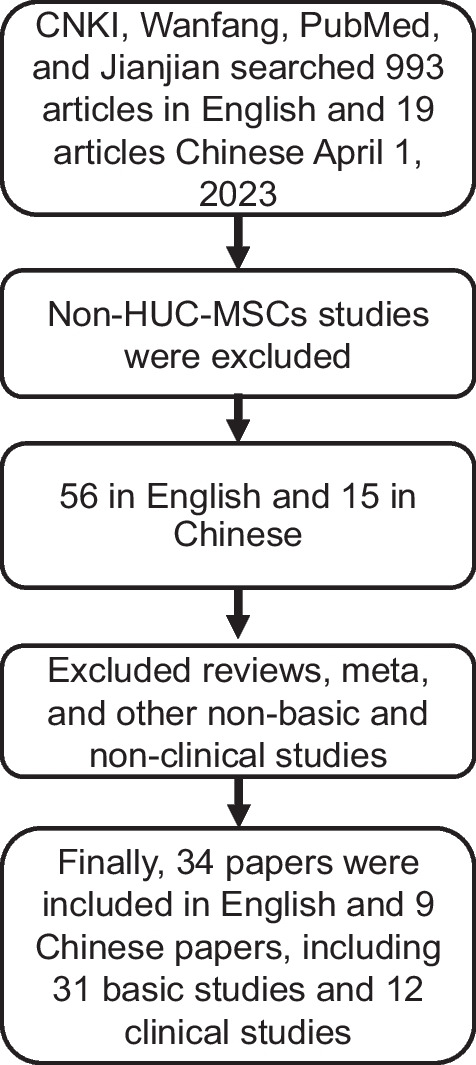
Flowchart for the identification and selection of studies

CNKI, Wanfang, PubMed, and articles related to the treatment of OA with HUC-MSCs since their publication until April 1, 2023, were collected, excluding non-basic and non-clinical studies such as reviews and meta-analysis. A total of 31 basic experimental studies and 12 clinical studies were included (Fig. 1). Systematically analyze the effects of HUC-MSCs on inhibiting inflammatory factors, promoting chondrocyte production, and current clinical treatment. Explain the relevant mechanisms and clinical effects of HUC-MSCs in treating OA. Analyze the advantages and disadvantages of HUC-MSCs in treating OA, as well as their safety in treating OA. Provide a systematic reference for the clinical use of HUC-MSCs in the treatment of OA. Contribute to guiding clinical treatment, reducing pain in OA patients, avoiding joint function loss, and improving quality of life.

### Study characteristics

This study systematically summarizes the mechanism of HUC-MSCs promoting chondrogenesis in the treatment of OA, as well as the clinical treatment results, and draws a table. And analyze the adverse events in the clinical treatment of HUC-MSCs. Provide a new approach for further clinical treatment of OA.

### The mechanism of action of HUC-MSCs on chondrocytes

#### Inhibiting OA inflammatory factors

**Fig. 2:**
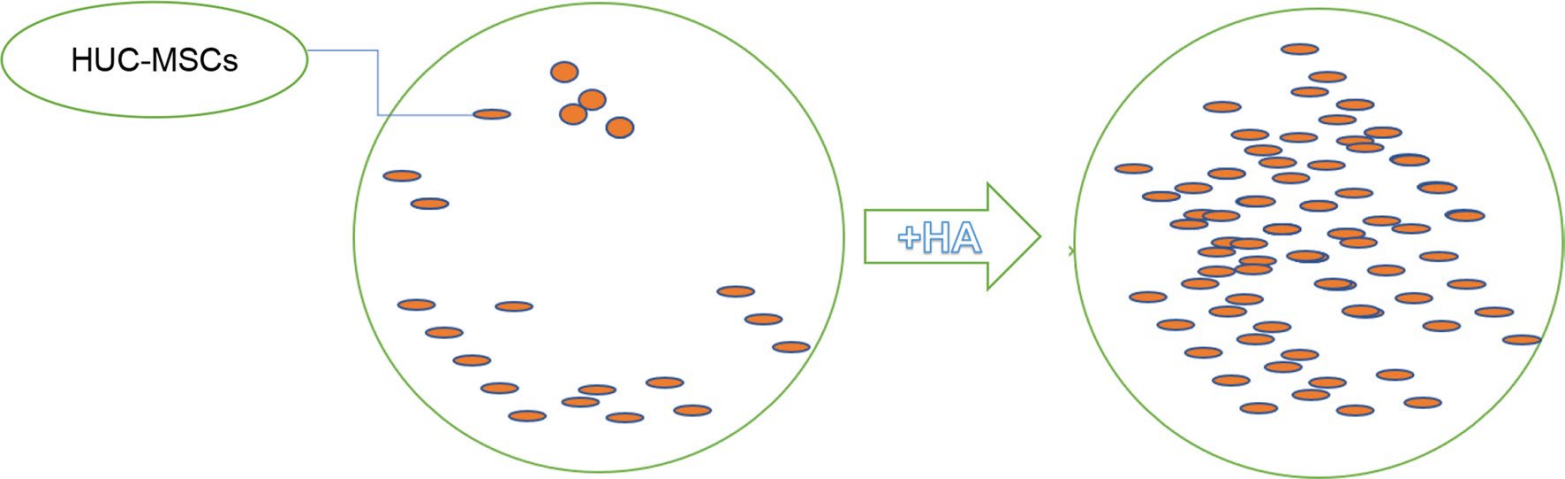
HA promotes the generationg of HUC-MSCs

The occurrence of OA is inseparable from the interaction of various inflammatory factors. HUC-MSCs inhibit the expression of matrix metalloproteinase 13 (MMP-13), collagen X (colx), and cyclooxygenase-2 (COX-2) in OA chondrocytes and enhance the proliferation of OA chondrocytes. Stimulate the expression of type II collagen (Col2), SRYbox9 (SOX9), and aggrecan, promoting the differentiation of HUC-MSCs into chondrocytes. Under certain induction conditions, HUC-MSCs observed osteogenic, chondrogenic, and adipogenic differentiation. HUC-MSCs improved the proliferation of OA chondrocytes and downregulated the expression of inflammatory cytokines, while OA chondrocytes promoted MSCs to differentiate into chondrocytes [5]. Human chondrocytes can withstand hypoxia and environmental conditions for more than 4 days and 10 days, respectively, after aggregation to form spherical bodies [6]. In a study of OA rats induced by sodium iodoacetate (MIA), local injection of HUC-MSCs significantly improved cartilage erosion and reduced Mankin score. The number of chondrocytes on the surface of articular cartilage was also significantly increased, and their catabolic markers, thrombospondin-5 (ADAMTS-5) and MMP-13, were also significantly reduced in the entire chondrocyte layer. Compared to a single injection, multiple HUC-MSCs injections better alleviated MIA induced infiltration of inflammatory cells on CD4 + Th cells and CD68 + macrophages, as well as synovial hyperplasia [7]. When combined with 4% sodium hyaluronate (HA), HUC-MSCs will proliferate faster and efficiently differentiate into chondrocytes (Fig. 2). In the pig OA model, transplantation of HUC-MSCs containing 4% HA not only improved macroscopic and microscopic histology, but also promoted the formation of cartilage in the treated joint [7]. When cultured in a bioreactor, the proliferation rate of HUC-MSCs is more than a thousand times that of bone marrow stem cells [9] (Fig. 2).

### Mechanism of HUC-MSCs differentiated cartilage cells

**Fig. 3:**
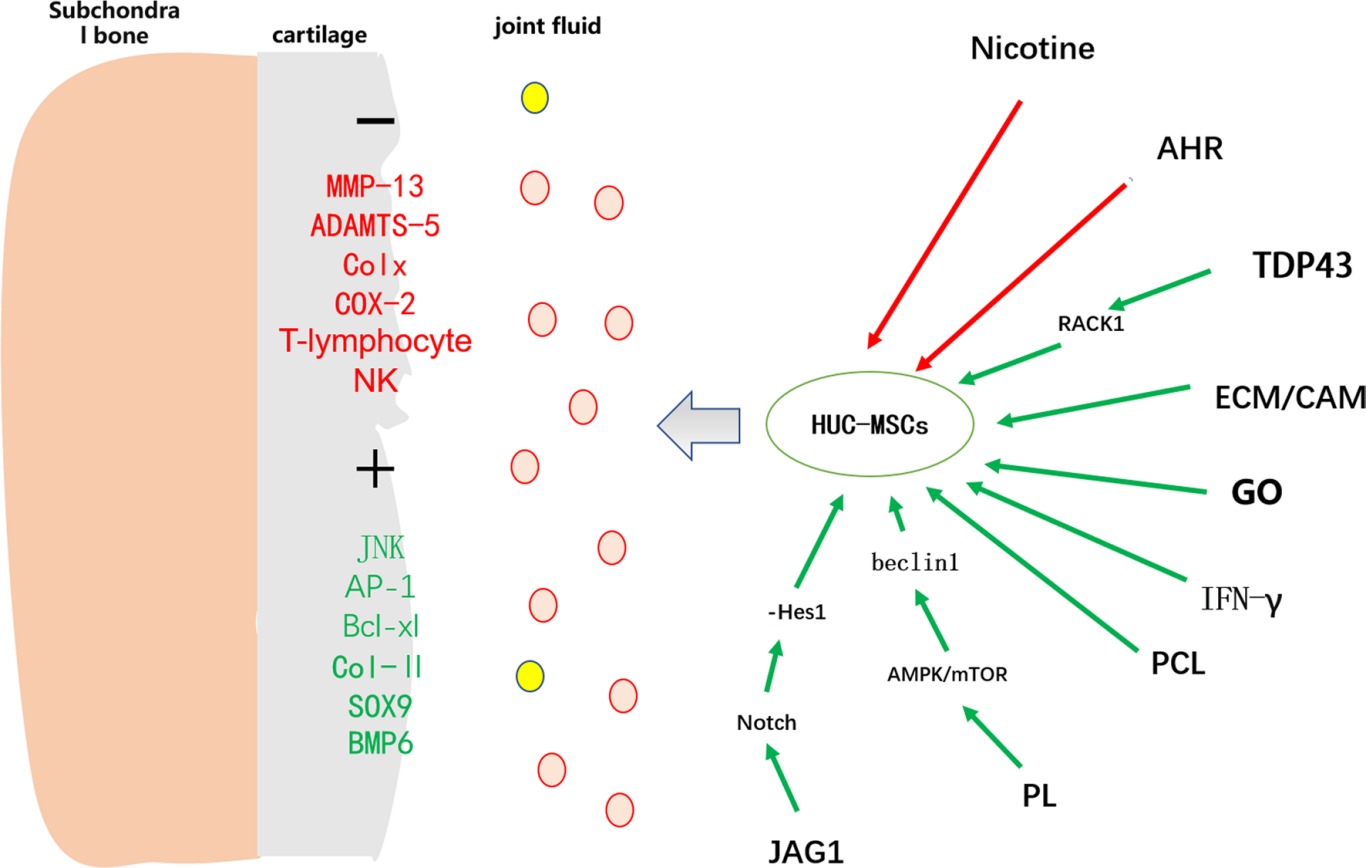
Red: inhibition, Green: promotion

The differentiation direction of HUC-MSCs is influenced by various conditions. The morphology of chondrocytes co-cultured with HUC-MSCs transfected with TDP43 Lentivirus changed significantly. The cell morphology becomes coarse, promoting chondrocyte proliferation and inhibiting cell apoptosis. After chondrocytes were co-cultured with TDP43 Lentivirus transfected HUC-MSCs, the expression of RACK1, JNK, AP-1, and Bcl xl genes was higher than that of chondrocytes co-cultured with non-transfected HUC-MSCs and vector Lentivirus transfected HUC-MSCs. High expression of TDP43 can activate the expression of RACK1, thereby regulating chondrocyte proliferation and apoptosis [10]. Compared with HUC-MSCs transfected with empty body Lentivirus, the morphology of chondrocytes co-cultured with untransfected HUC-MSCs did not change, and they were spindle shaped adherent growth. The same research results indicate that HUC-MSCs stimulate local endogenous chondroprogenitor cell differentiation through TSP-2, ultimately leading to cartilage regeneration [11]. Extracellular matrix (ECM) derived from cartilage can promote the transformation of HUC-MSCs into chondrocytes and promote cartilage formation. However, the role of HUC-MSCs stimulated by ECM in OA is still unclear. Cartilage acellular matrix (CAM) is a cartilage-derived ECM used to promote chondrogenesis in HUC-MSCs. Analyze the ability of HUC-MSCs to differentiate into cartilage using chondrogenic markers (aggrecan, type II collagen, and SOX9) and bone morphogenic protein 6 (BMP6). CAM treatment significantly increased the expression of chondrogenic markers and BMP6 in HUC-MSCs. In addition, HUC-MSCs and CAM treatment not only enhanced the synthesis of proteoglycans and Col2, but also enhanced the anti-inflammatory effects of rabbit joints and synovial fluid in the rabbit cruciate ligament transection (ACLT) model. The involvement of HUC-MSCs and BMP6 was also detected in rabbit cartilage tissue. Therefore, BMP6 can induce HUC-MSCs to generate CAM [12]. The combination of various biomaterials and HUC-MSCs is gradually being used to treat OA. HUC-MSCs loaded with graphene oxide (GO) particles lubricant can promote the secretion of chondrocytes in OA animal models, reduce the level of intra-articular inflammation [13], improve subchondral bone osteoporosis, and promote cartilage repair [14]. The electrospun polycaprolactone (PCL) nanofiber network combined with HUC-MSCs for cartilage tissue engineering may also have an impact on OA. Compared with HUC-MSCs cultured on electrospun nanofiber net, articular chondrocytes isolated from human osteoarthritis joints have higher production of glycosaminoglycan and higher expression of cartilage related genes in HUC-MSCs cultured on basic medium. In addition, the presence of sulfated proteoglycans and ColII was observed on both types of cell cultures. This effect is due to the structure of PCL or the inherent cartilage differentiation potential of HUC-MSCs [15]. In vitro research results indicate that platelet lysates (PL) significantly promote the proliferation of HUC-MSCs by upregulating related genes/proteins and activating Beclin1-dependent autophagy through the AMPK/mTOR signaling pathway. In vivo data indicate that the combination of PL and HUC-MSCs has a significant synergistic effect on OA. Overall, the beneficial effects and mechanisms of PL on HUC-MSCs were identified, and PL was indicated as an adjuvant for HU-CMSCs in treating OA [16]. The combination of soluble Jagged1 (JAG1) peptide and HUC-MSCs enhances the survival rate and cartilage differentiation of HUC-MSCs, reduces local inflammation, and further promotes its therapeutic effect. The inhibition of Notch targeting Hes1 expression by JAG1 can enhance the survival and cartilage differentiation of HUC-MSCs, thereby enhancing their therapeutic potential for cartilage regeneration [17]. In the study of UCB MSCs combined with 4% HA hydrogel composite on articular cartilage, the finding that transplanted cells disappear at the defect site shows that the paracrine interaction between UCB MSCs and host cells plays an important role in cartilage repair. UCB MSCs and 4% HA hydrogel composite may be a new treatment for full-thickness cartilage defects [18] (Fig. 3).

### Inhibits the ability of HUC-MSCs to differentiate cartilage

Some substances inhibit the ability of HUC-MSCs to differentiate cartilage. In an OA patient and animal experiment, the synovial fluid of OA patients activated aromatic hydrocarbon receptor (AHR) expression in HUC-MSCs and inhibited cartilage differentiation. Inhibition of AHR expression relieves this inhibition [19]. Similarly, studies looking at the effect of nicotine on HUC-MSCs found that from the 3rd day of treatment, nicotine significantly impaired the proliferation of HUC-MSCs, but nicotine did not change the viability of HUC-MSCs, but the content of proteoglycans in the control group was richer than in the nicotine group, but there was no difference in total collagen content. The mRNA expression of SOX9, ColII, and aggrecan glycans in the control group was higher than that in the nicotine group. Calcium channels are initiated by α7 nicotinic acetylcholine receptor (α7nAChR) after nicotine leads to calcium (Ca2 +) influx into HUC-MSCs to affect proliferation, so nicotine has adverse effects on the proliferation of HUC-MSCs and cartilage differentiation, which may be impaired by α7nAChR mediation [20]. In vitro studies have shown that after stimulation by pro-inflammatory factors (IFN-γ), HUC-MSCs consume tryptophan through the kynurenine pathway by upregulating the mRNA of the immunomodulatory molecule indolamine 2,3-dioxygenase (IDO), inhibit the proliferation of pro-inflammatory factors such as T cells and NK cells, and inhibit the inflammatory response [21].

### HUC-MSCs-EV for knee osteoarthritis

#### EV inhibits OA inflammatory factors

Exosomes (EVs) are nanoscale membrane vesicles such as lipids, microRNAs, lncRNAs, and specific proteins between 40 and 100 nm [22]. HUC-MSCs-EV are produced by the endomorphic body network and pass through many different pathways such as the ESCRT pathway acting on exosomal substances [23], inhibiting inflammatory mediators and MMP activity and promoting chondroprotective effects of anti-inflammatory cytokines on OA. It has become an excellent choice for the use of cell-free therapy in degenerative diseases through immunomodulation and tissue regeneration [24]. EVs have immunomodulatory and anti-inflammatory effects in various inflammatory diseases and tissue damage [25]. Most of the anti-inflammatory factors TSG-6 and IL-1RA in OA rats are derived from chondrocytes, and HUC-MSCs affect the OA microenvironment through EV and regulate inflammatory response [26]. When HUC-MSCs were injected intra-articularly, an increase in the expression of TNF-α-stimulated gene/protein 6 (TSG-6) in articular cartilage was observed and a decrease in MMP and ADAMTS-5 was observed. In addition, the expression of anti-calcitonin gene-related peptides in OA rats was also significantly reduced, indicating that it had an inhibitory effect on the central sensitizing component of pain [27]. There are many types of EVs. It has been found that the disappearance of transplanted cells at the defect site by intra-articular injection of UCB-MSCs overexpressing miR-140-5p combined with 4% HA hydrogel composite showed that EV can promote the expression levels of ColII, aggrecan and SOX9 and inhibit the expression levels of mRNA and protein of NLRP3, caspase-1, MMP-13, and ADAMTS-5, inhibits cartilage degeneration in osteoarthritis rats, and improves cartilage repair function [28]. By miR-1208 targeting combined with METTL3 (METTL3 can play a protective role against OA), the m6A level of NLRP3 mRNA was reduced, the expression of ColII and aggrecan was increased, the overexpression of ADAMTS-5 and MMP-13 in the knee joint of mice was inhibited, the secretion of pro-inflammatory factors was reduced, the progression of OA was slowed down, osteophyte production was reduced, the degradation of cartilage ECM was reduced, and the OA progression of mice was alleviated [29]. In vitro cell line analysis showed that EVs enhanced chondrocytes proliferation and migration while inhibiting apoptosis. HUC-MSCs produce nutritional effects on endogenous cell populations by secreting a large number of growth factors and cytokines, as well as immunomodulatory and anti-inflammatory molecules, EVs, etc.

#### EV acts on the signaling pathway of OA cartilage

Intra-articular injections of HUC-MSCs-EV can mitigate cartilage destruction and matrix degradation. EV maintains the chondrocytes phenotype by increasing ColII, synthesis and decreasing ADAMTS-5 expression through IL-1β. Later, in animal experiments, it was shown that intra-articular injection of HUC-MSCs-EV successfully prevented cartilage destruction in DMM models [30]. Intraperitoneal injections into OA animal models in HUC-MSCs-EV are made twice, one month after treatment. The results showed that EV is an effective treatment for reducing chemokines and cytokines in the serum of OA animals and aging-related secretory phenotyping (SASP). EVs are statistically significant compared to HUC-MSCs alone. Derived EVs show higher therapeutic potential. Systemic inflammation is improved by inactivation of the ERK1/2-AKT pathway in the in vivo OA model. EV treatment with mesenchymal stem cells previously modified to contain miR-21 antagonists is more effective at reducing systemic inflammation in age-related diseases than miR-21 in binding HUC-MSCs themselves. In addition, MSC-miR-21-derived EVs can modulate the ERK1/2 family to exert anti-inflammatory effects in the OA model via SDC1 [31].

Most EVs have limited access resources and are at risk of host rejection and immune response. The EVs of HUC-MSCs have the advantages of easy availability, minimal immune rejection, and good immunomodulatory effect. Although the exact mode of action of EV treatment for OA is unknown, there are various types of EVs, and the effect of different EVs on OA cartilage is also very different, and compared with normal cartilage, treatment with exosome MSC-92a-3p-Exos promotes HUC-MSCs cartilage proliferation and stromal gene expression. Conversely, treatment with MSC-aanti-miR-92a-3p-Exos inhibits cartilage differentiation and reduces cartilage matrix synthesis by enhancing the expression of WNT5A. Further studies showed that miR-92a-3p inhibited the activity of reporter gene constructs containing 3′-untranslated regions (3’-UTR) and inhibited the expression of WNT5A, regulating cartilage development and homeostasis. Exosome miR-92a-3p may act as a Wnt inhibitor and have shown potential as a disease-modifying osteoarthritis drug [32].

In animal studies, EVs at 15 μg/mL showed maximum proliferation and migration capacity. This effect is caused by maintaining cartilage homeostasis, as evidenced by an increase in COLII and a decrease in MMP-13 and ADAMTS-5. M1 macrophage markers (CD14) were significantly reduced for HUC-MSCs and HUC-MSC-EV, while M2 macrophage markers (CD206 and IL-10) were increased. Cartilage repair-related proteins are more abundant in EVs. Compared with HUC-MSCs, upregulated proteins in EVs are mainly involved in the regulation of immune effector processes, extracellular matrix tissue, PI3K-AKT signaling pathway, and Rap1 signaling pathway. However, the disadvantages of EV treatment, such as the additional pain caused by multiple injections, the high cost of isolation methods, and the low enrichment of EVs, require further research [33].

**Table:** Clinical application of HUC-MSCs in the treatment of OA

Essay	Sample size (example)	Observe the metrics	Duration of follow-up (yea)	Injection dose (pcs/ml)	Results
Günay [34]	10	VAS, WOMAC, and Lequesne scored 36 short health surveys. On MRI imaging, tumor necrosis factor α, interleukin-1β, adiponectin, resistin, and interleukin-6	1 year	1 × 10^8^/1 ml	VAS, WOMAC, and Lequesne scores were lower, with 36 short health surveys having higher average scores. On magnetic resonance imaging, it was found that the thickness of cartilage was increased. Tumor necrosis factor α, interleukin-1β, adiponectin, resistin, and interleukin-6 levels were significantly elevated
Suh [35]	43	Patients with HOT with ICRS class IV and medial femoral condyle cartilage defects greater than 200 mm2 were included. Clinical, Hospital for Special Surgery (HSS), International Knee Literature Committee (IKDC), and Lysholm scores were assessed 18 months postoperatively	1.5 years	7.5 × 10^6^/1.5 ml	The clinical efficacy and JSW of HUC-MSCs in HTO patients were better than those of microfractures
Lim [36]	144	International Knee Documentation Committee (IKDC) and Lysholm score	5 years	7.5 × 10^6^/1.5 ml	In older patients with symptomatic, large, full-thickness cartilage defects with or without osteoarthritis, HUC-MSCs-HA implantation improves cartilage grade at arthroscopy and improves pain and function over 5 years compared with microfractures
Song [37]	125	Imaging, mechanical axes, and JSW	3 years	7.5 × 10^6^/1.5 ml	HUC-MSCs in combination with HTO is an effective treatment for patients with medial ventricular osteoarthritis and varus deformity.
Song [38]	25	ICRS, histological assessment; VAS score, WOMAC, KDC score	1–2 years	7.5 × 10^6^/1.5 ml	ICRS raised by 1 level. In patients over 60 years of age with OA, HUC-MSCs implantation achieves satisfactory cartilage regeneration and shows satisfactory clinical results
Dilogo [39]	29	Age > 40 years, varus deformity > 5°, (ICRS) grade IV articular cartilage damage > 4 cm^2^	0.4 year	1 × 10^7^/2 ml	HUC-MSCs achieve maximum effect after 6 months of injection
Matas [40]	18	IKDC score, VAS, WOMAC score	1 year	2 × 10^7^/3 ml	In phase I/II clinical trials, repeated HUC-MSCs injection strategies resulted in good safety and improved clinical outcomes for treating long-term pain in patients with knee OA
Park [41]	9	MRI, arthroscopy to assess cartilage regeneration	7 years	0.5 × 10^7^/ml	Histology after 1 year shows hyaline cartilage. MRI after 3 years showed that the regenerative cartilage persisted. No bone changes were found within 7 years
Wang [42]	18	Kellgren–Lawrence Level I–IV examination. MRI findings	0.5 year	(2–3) × 10^7^/mL	Intra-articular injections of HUC-MSCs for OA significantly improve joint function and quality of life
Yang [43]	28	WOMAC, VAS SCORE, SF-13, OVERALL PATIENT ASSESSMENT, AND RHEUMATOLOGY COMMITTEE (OMERACT) OARSI, WOMAC, KNEE MRI EVALUATION AND BLIND ASSESSMENT	1 year	1.5 × 10^7^/ml	The effect begins 1 month after injection, and the therapeutic effect can last for 6 months
Chung [44]	93	Safety, VAS score, International Knee Documentation Committee (IKDC) subjective score, MRI, histological evaluation	1.7 years	0.5 × 10^7^/ml	HUC-MSCs knee injections can treat severe OA
Samara [1]	16	Clinical treatment was evaluated using SF-36 scale, Lysholm score, and WOMAC score	2 years	0.6 × 10^6^/ml	Sodium hyaluronate provides faster, more significant, and longer-lasting relief

#### HUC-MSCs have long-term effects in patients with OA

With the remarkable results of HUC-MSCs in the treatment of OA in basic trials, HUC-MSCs have gradually begun to be studied in clinical practice. Most of the HUC-MSCs used in clinical practice are allogeneic stem cells derived from umbilical cord blood with sufficient growth factors and cytokines for regeneration. Clinical studies of high tibial osteotomy (HTO) versus HUC-MSCs have reported good clinical outcomes and cartilage regeneration, producing more hyaline cartilage than microfractures (MFx) in patients over 50 years of age. The current study is consistent with these results and has good clinical outcomes and an increase in joint space (JSW) in elderly patients. In a multicenter randomized study of HUC-MSCs and MFx, HUC-MSCs showed better cartilage regeneration in clinical outcomes 48 weeks postoperatively. However, after HUC-MSCs treatment, clinical outcomes are better 3–5 years after surgery in patients with OA. HUC-MSCs can enhance cartilage regeneration in HTO patients with cartilage defects greater than 200mm^2^ [35]. At 48 weeks, the International Society for Cartilage Repair (ICRS) grade improvement was 1 grade higher in the umbilical cord-derived mesenchymal stem cells combined with 4% hyaluronic acid (HUC-MSCs-HA) compared with the microfracture group; the overall histological assessment score of the HUC-MSC-HA group was also better than that of the MFx group. Clinical outcomes in the HUC-MSCs-HA group improved significantly at 3- to 5-year follow-up. Studies have also shown the greatest improvement in knee function after 6 months of follow-up [39]. This new application of HUC-MSCs-HA may be applicable to OA patients. HUC-MSCs-HA may be an alternative to single-compartment knee replacement for patients who wish to preserve the joint and maintain an active lifestyle. In patients with OA more than 7 years after treatment, improved clinical outcomes did not deteriorate significantly. Cartilage repair by HUC-MSCs-HA can lead to sustained clinical improvement within 5 years of intervention, which is highly beneficial for joint protection. Large full-thickness cartilage defects can also be regenerated for treatment [36]. The clinical impact of HUC-MSCs treatment exceeds the expected placebo effect described by most treatments for knee osteoarthritis [45]. In phase I/II randomized, controlled, triple-blind trials of HUC-MSCs followed by 6 or 12 months follow-up, no changes were found, possibly due to a slight effect on imaging and a low initial WORMS score, although studies have shown the preventive effect of MSC cell therapy on OA development, so long-term 2-year data were collected. The control group was found to have an increased chance of disease progression at 2 years of follow-up [40].

#### Factors influencing HUC-MSCs in the treatment of OA

The formation of OA is affected by many factors, obesity, age, work environment, and activity level which are all important factors affecting the clinical outcomes of OA patients. However, clinical observations in patients with OA treated with HUC-MSCs showed that clinical scores at 2 years after injection improved compared with 1 year, regardless of obesity. Age is an important factor influencing clinical outcomes. < 65-year-old OA patients outperformed the 6 ≥ 5-year-old group in IKDC scores at 1 and 2 years and VAS at 2 years. And the younger the age, the higher the improvement in IKDC, VAS, and WOMAC scores in patients with OA. The ≥ 65-year-old group will also improve over time. In addition, chondrogenic activity of HUC-MSCs is also reduced in cultures from patients with advanced OA [44]. The size of the cartilage defect is also another factor affecting clinical outcomes, but is not associated with 1- and 2-year IKDC, VAS, and WOMAC scores [38]. The number of stem cells regarding defective size has not been standardized, and further investigation is needed, because the results of another study showed that the advanced age or older cartilage damage in patients had no significant effect on the clinical outcome of HTO and implantation of HU-CMSCs in OA patients [37]. However, local injection of HUC-MSCs in the treatment of degenerative knee osteoarthritis had a significantly better duration of efficacy after treatment than local injection of sodium hyaluronate [42].

#### Safety analysis of HUC-MSCs in the treatment of OA

HUC-MSCs treated with OA patients had fewer adverse effects, and no significant differences in overall or treatment-specific adverse events were observed between the HUC-MSCs group and the microfracture group in the initial 48-week clinical trial and 60-month follow-up survey. Nor did participants drop out of the study due to adverse events. Three serious adverse events (SAEs) occurred in 3 participants treated with HUC-MSCs in combination with HA, compared with 1 participant in the microfracture group with 2 SAEs in the first 48 weeks. Pain at the treatment site in the HUC-MSCs-HA group is thought by the investigators to be related to the treatment modality. At 60 months of follow-up, 8 SAEs occurred in 7 participants in the HUC-MSCs-HA group and 7 in 5 participants in the microfracture group. The researchers believe that none of the SAEs were associated with treatment. Among them, OA patients who underwent total knee replacement and high tibial osteotomy were considered to have a natural history of osteoarthritis, rather than implantation of the HUC-MSCs-HA complex. One death due to myocardial infarction (41 months after intervention) was reported in the HUC-MSCs-HA group. In an additional [45], participants treated with HUC-MSCs-HA, no immune response was observed. Also in other studies, in clinical trials of seven patients with Kellgren–Lawrence class III osteoarthritis and ICRS class IV cartilage defects [46]. Five participants reported mild to moderate adverse events, but no serious adverse events such as arthralgia, back pain, bladder dilation, and elevated levels of anti-thyroglobulin antibodies. Only those classified as “increased susceptibility to infection” according to WHO criteria were identified as treatment emergency adverse events (TEAEs). An internal medicine specialist judged that it did not require additional treatment and returned to normal automatically. Prospective follow-up was conducted for at least one year in patients who received HTO therapy and HU-CMSCs implantation with full-thickness cartilage injury in the knee with osteoarthritis. Ninety-three patients were followed for an average of 1.7 years (range 1.0–3.5 years). The average size of cartilage lesions was 6.5 cm^2^ (range 2.0–12.8 cm^2^). After HUC-MSC implantation, no persistent effusion, synovitis, local rash, or local erythema were found. Some patients have knee swelling for up to 1 month after surgery and require pain medication [43]. At 7-year follow-up, no specific adverse effects were observed in 6 participants. During follow-up, no participants underwent additional knee surgery or knee replacement due to knee pain, exacerbation, or impaired function. Only one participant died during the long-term extension period because she died of frail old age 6 years after transplantation at the age of 76. Nor were any unusual findings indicating rejection or infection. None of the participants had substantial permanent morbidity, and none of the participants terminated the study prematurely after transplantation. Although they use allogeneics, HUC-MSCs result in less rejection due to their low immunogenicity and immunomodulatory activity. Another notable finding was that the improvement in pain and function 24 weeks after transplantation did not deteriorate significantly within 7 years. Participants in the study were patients with K-L III OA but did not undergo any additional knee surgery or knee replacement surgery during the 7-year follow-up period after transplantation [41]. Similarly, a meta-analysis evaluating the safety of HUC-MSCs over 15 years found that HUC-MSCs administration was significantly associated with transient fever, adverse events at the site of administration, constipation, fatigue, and insomnia. Despite these barriers, there is evidence that HUC-MSCs injections have a beneficial effect in the treatment of knee osteoarthritis [47].

In a randomized semi-blind experiment, the symptoms and signs of self-perceived abnormalities occurred in the trial, mainly including increased blood pressure, dizziness, insomnia, hot flashes, epigastric discomfort, nausea, transient joint pain aggravation (≤ 24 h), influenza symptoms, etc. Among them, 2 cases (13.3%) occurred in the 2 injections of HUC-MSCs (15 cases), of which 1 case was mildly untreated and self-healed, and 1 case had moderate increase in blood pressure (150/100 mmHg) and was treated symptomatically to improve with antihypertensive treatment. A total of 3 adverse events occurred in 4 injections of HUC-MSCs (13 cases) (incidence 21.4%), all of which were mild untreated self-healing. There were no significant differences in the incidence of adverse events between 2 and 4 injections and the incidence of individual adverse events. Laboratory tests showed 2 injections of HUC-MSCs with abnormal liver function (mild elevation of ALT/AST) in 2 cases (incidence 13.3%), which were mildly elevated and normal without treatment. Liver dysfunction (mildly elevated ALT/AST) in 4 injections of HUC-MSCs was mild in 1 case (incidence 13.3%), and untreated re-examination was normal [43]. Treatment was well tolerated. Function and pain improved significantly at 12 and 48 months, MRI scans showed statistically significant improvements in cartilage loss, osteophytes, bone marrow lesions, effusions, and synovitis at 12 months, and subchondral sclerosis was very significant.

In addition, a total of 29 in vivo studies were identified and analyzed in the PubMed and Scopus databases. Studies reported outcomes in EV delivery and uptake in vivo; improve cartilage morphology, histology, and biochemical outcomes; enhance subchondral bone regeneration; and improvement of painful behavior after EV treatment. Studies have demonstrated that EV treatment is safe, as no significant complications were found in all included studies. EV administration is an effective cell-free therapy for the relief of OA. However, further research is needed to confirm the therapeutic potential of EVs and to determine the standard regimen for OA based on EV therapy.

## Discussion

The onset of OA patients is affected by a variety of factors, which eventually lead to the loss of cartilage in the joints, the formation of osteophytes, the loss of normal knee mobility, and pain and discomfort, which seriously affects the quality of life. Articular cartilage is mainly composed of chondrocytes and extracellular matrix, because cartilage does not contain blood vessels and nervous system, and the required nutrients depend on the synovial fluid of the joint and the arterial layer branches around the synovial membrane to maintain the normal synthesis and metabolism of chondrocytes. Articular cartilage exists at the junction of adjacent bones, its surface is smooth and elastic, can play a buffer role when the joint moves, and is indispensable for maintaining the normal function of the joint, but this also makes the joint cartilage extremely vulnerable to destruction. Therefore, preventing the loss of articular cartilage is essential to prevent the development and development of OA.

HUC-MSCs are a relatively novel source of mesenchymal stem cells for the treatment of OA. HUC-MSCs extracted from the umbilical cord have a greater proliferative capacity and are not subject to the same ethical controversy as human embryonic stem cells. Mesenchymal stem cells derived from the umbilical cord offer multiple advantages in osteoarthritis cartilage repair over other sources of mesenchymal stem cells. First, newborn umbilical cord specimens are abundant and can be provided in large quantities. Second, there is no trauma or pain to the individual during the collection process. Third, proliferation is rapid and non-tumorigenic. Fourth, as a pluripotent stem cell, due to its multi-directional differentiation potential, it can differentiate into a variety of cells under certain conditions, participate in the repair of tissue damage, and have higher inducibility. Fifth, a large number of cytokines can also be secreted through paracrine function to participate in the inflammatory immune regulation process, making HUC-MSCs ideal cells for the treatment of OA cartilage damage. And compared with bone marrow mesenchymal stem cells, UHC-MSCs show significantly higher chondrogenesis potential and relatively lower osteogenic and lipogenic capacity. In the process of cartilage formation, the expression of cell proliferation, adhesion molecules, signaling molecules, and chondrogenesis-specific genes of UHC-MSCs is significantly increased. For therapeutic effect, in arthritis models, UHC-MSCs can increase the synthesis of ColII and reduce proteoglycan expression of pro-inflammatory cytokines. And HUC-MSCs contain a large number of growth factors, cytokines, hyaluronic acid, and extracellular vesicles [47]. Through cytokine profiling, IL-10, ICAM-1, and TGFb1 were also upregulated more pronounced in UHC-MSCs. UHC-MSCs do have a particular advantage over bone marrow mesenchymal stem cells in terms of cartilage regeneration [48]. Under anaerobic conditions, undifferentiated HUC-MSCs can produce better extracellular matrix (ECM). Compared to bone marrow mesenchymal stem cells, HUC-MSCs produce higher amounts of type II collagen, specifically the IIB subtype, and also produce type I collagen and Htra1 collagen [49]. The biological advantages of HUC-MSCs include high proliferation rate and clonality, anti-aging, and excellent anti-inflammatory effects. In recent years, drugs mixed with HUC-MSCs with hyaluronic acid have been widely used in clinical settings. HUC-MSCs have the advantage of low immunogenicity, and the cartilage formed can display a hyal-like histological morphology [50]. HUC-MSCs can also inhibit the expression of calcinogen 11 (CDH11) in rheumatoid arthritis (RA) via fibroblast-like synovial cells (FLS). This mechanism may help improve arthritis [51].

The number of injections used in the treatment has also not been clearly defined in the current study, and in the ACLT-induced rat model, single versus two injections of 1 ml at a concentration of 1 × 10^6^ (cells/ml) were compared and followed for 12 weeks. Serial sections of the knee were subjected to histological, immunohistochemical, and TUNEL analysis. Results: Transplantation of HUC-MSCs significantly reduced the development of OA-induced by ACLT surgery. Significantly promotes ACLT-induced synthesis of chondrocytes extracellular matrix synthesis (aggrecan). TUNEL analysis showed that HUC-MSCs treatment significantly protected chondrocytes from apoptosis. No significant differences were observed between single and repeated injections. In vitro HUC-MSCs show good cartilage-forming potential, although HUC-MSCs exhibit excellent proliferative capacity. However, single or two injections of HUC-MSCs significantly inhibited the progression of ACLT-induced OA rats with similar effects, and HUC-MSCs reduced ACLT-induced apoptosis of arthritic chondrocytes [52]. Similarly, HUC-MSCs transplantation for severe OA can reduce joint pain and improve joint function more quickly, significantly and durably than sodium hyaluronate, and the efficacy of 4 injections is better than 2 injections. Therefore, multiple injections may produce better results.

HUC-MSCs are well tolerated, effective, and safe in the treatment of patients with OA. Repeated dosing and higher concentrations led to better clinical improvement. A reduction in cartilage loss was observed in some HUC-MSCs trials. No serious adverse effects were recorded. Treatment in KOA-KL II-III may be more effective [53]. In a non-randomized, open-label, multicenter trial, Ashim Gupta et al. came to the same conclusion [54].

## Conclusion

HUC-MSCs can reduce inflammatory factors such as MMP-13, ADAMTS-5, IL-1β, IL-1, IL-1, IL-6, TNF-α, M1 polarization in OA and protect cartilage damage. At the same time, the synthesis of ColII, SOX9, aggrecan, etc., promotes cartilage synthesis, promotes cartilage synthesis through P13/Akt, mTOR, Notch, and other cell signaling pathways, and acts on THP-1 to induce the conversion of M1 to M2 to reduce the level of OA inflammation. Clinically, HUC-MSCs produce more hyaloid cartilage than MFx in patients older than 50 years of age. Better cartilage regeneration was shown in clinical outcomes 48 weeks postoperatively. These studies also suggest that HUC-MSCs can enhance cartilage regeneration in HTO patients with cartilage defects greater than 200mm2, and that treatment in KOA-KL II-III may be more effective than sodium hyaluronate for the treatment of severe cartilage damage. In terms of safety, it is mainly pain-based and can recover on its own, without intervention and treatment, which is relatively safe.

### Further study

There are many factors in the treatment of OA by HUC-MSCs, such as the extraction, storage, temperature, and use of preservatives or catalysts for HUC-MSCs, which can affect the effectiveness of treatment. There is also no clear definition of the dose of OA treatment, and 7.5 × 106 cells/1.5 ml are used in existing articles, and the safety profile is better. Therefore, we are far from knowing the optimal conditions for using HUC-MSCs as a therapeutic tool in preclinical and clinical trials of OA. Although many clinical, radiographic, and histological studies have shown that HUC-MSCs improve knee chondrocytes. More basic research and multicentre randomised clinical studies are needed to determine the significant clinical impact on OA.

## Data Availability

This study collected CNKI, Wanfang, PubMed, and articles related to the treatment of OA with HUC-MSCs since their publication, excluding non-basic and clinical studies such as reviews and meta-analysis. A total of 31 basic experimental studies and 12 clinical studies were included. Systematically analyze the effects of HUC-MSCs on inhibiting inflammatory factors, promoting chondrocyte production, and current clinical treatment.

## References

[1] Samara O, Jafar H, Hamdan M (2022). Ultrasound-guided intra-articular injection of expanded umbilical cord mesenchymal stem cells in knee osteoarthritis: a safety/efficacy study with MRI data. Regen Med.

[2] Shi D, Clement ND, Bhonde R, Ikegawa S, Mascarenhas VV, Di Matteo B, Indelli PF, Kourkoumelis N, Rodríguez-Merchán EC, Sheykhhasan M, Fu FH, Vaz MA, Kong J, Azantsa BG, Ye C, Halabchi F, Cornish SM, Hausmann LR, Campos ALS, Lopes de Jesus CC, Jorgensen C, Ilieva EM, Wang W, Villarreal-Martínez L, Ahn H, Shirinsky IV, Cheung C, Knutsen G, Petersen W, Lane NE, Cai H, Xu W, Wu J, Zhang Y, Lu J, Jiang Q (2019). Society for translational medicine-expert consensus on the treatment of osteoarthritis. Ann Joint.

[3] Xing D, Wu J, Wang B (2020). Intra-articular delivery of umbilical cord-derived mesenchymal stem cells temporarily retard the progression of osteoarthritis in a rat model. Int J Rheum Dis.

[4] Jang S, Lee K, Ju JH (2021). Recent updates of diagnosis, pathophysiology, and treatment on osteoarthritis of the knee. Int J Mol Sci.

[5] Wang H, Yan X, Jiang Y (2018). The human umbilical cord stem cells improve the viability of OA degenerated chondrocytes. Mol Med Rep.

[6] Jiang B, Fu X, Yan L (2019). Transplantation of human ESC-derived mesenchymal stem cell spheroids ameliorates spontaneous osteoarthritis in rhesus macaques. Theranostics.

[7] Tong W, Zhang X, Zhang Q (2020). Multiple umbilical cord-derived MSCs administrations attenuate rat osteoarthritis progression via preserving articular cartilage superficial layer cells and inhibiting synovitis. J Orthop Transl.

[8] Wu KC, Chang YH, Liu HW (2019). Transplanting human umbilical cord mesenchymal stem cells and hyaluronate hydrogel repairs cartilage of osteoarthritis in the minipig model. Ci Ji Yi Xue Za Zhi..

[9] Gupta A, Maffulli N, Rodriguez HC, Carson EW, Bascharon RA, Delfino K, Levy HJ, El-Amin SF (2021). Safety and efficacy of umbilical cord-derived Wharton’s jelly compared to hyaluronic acid and saline for knee osteoarthritis: study protocol for a randomized, controlled, single-blind, multi-center trial. J Orthop Surg Res.

[10] Perry J, Roelofs AJ, Mennan C, McCarthy HS, Richmond A, Clark SM, Riemen AHK, Wright K, De Bari C, Roberts S (2021). Human mesenchymal stromal cells enhance cartilage healing in a murine joint surface injury model. Cells.

[11] Huang Y, Huang Q, Liu Y (2020). Proliferation and apoptosis of human umbilical cord mesenchymal stem cells and chondrocytes after co-culture of TDP43 lentiviral vector. Chin Tissue Eng Res.

[12] Jeong SY, Kim DH, Ha J (2013). Thrombospondin-2 secreted by human umbilical cord blood-derived mesenchymal stem cells promotes chondrogenic differentiation. Stem Cells.

[13] Jeon HJ, Yoon KA, An ES (2020). Therapeutic effects of human umbilical cord blood-derived mesenchymal stem cells combined with cartilage acellular matrix mediated via bone morphogenic protein 6 in a rabbit model of articular cruciate ligament transection. Stem Cell Rev Rep.

[14] Liu A, Wang P, Zhang C (2020). Graphene oxide nanoscaffolds cultured umbilical-derived MSCs regulating the repair of joint microenvironment on cartilage. Chin J Integr Surg.

[15] Liu A, Chen J, Zhang J (2022). Intra-articular injection of umbilical cord mesenchymal stem cells loaded with graphene oxide granular lubrication ameliorates inflammatory responses and osteoporosis of the subchondral bone in rabbits of modified papain-induced osteoarthritis. Front Endocrinol (Lausanne).

[16] AlvesdaSilva M, Martins A, Costa-Pinto AR (2017). Electrospun nanofibrous meshes cultured with Wharton’s jelly stem cell: an alternative for cartilage regeneration, without the need of growth factors. Biotechnol J..

[17] Yan L, Zhou L, Yan B (2020). Growth factors-based beneficial effects of platelet lysate on umbilical cord-derived stem cells and their synergistic use in osteoarthritis treatment. Cell Death Dis.

[18] Sun J, Luo Z, Wang G (2018). Notch ligand Jagged1 promotes mesenchymal stromal cell-based cartilage repair. Exp Mol Med.

[19] Park YB, Ha CW, Kim JA (2017). Single-stage cell-based cartilage repair in a rabbit model: cell tracking and in vivo chondrogenesis of human umbilical cord blood-derived mesenchymal stem cells and hyaluronic acid hydrogel composite. Osteoarthr Cartil.

[20] Wang X (2021). Role of aromatic hydrocarbon receptor-mediated cartilage differentiation of human umbilical cord-derived mesenchymal stem cells in cartilage injury repair in rats with osteoarthritis.

[21] Yang X, Qi Y, Avercenc-Leger L (2017). Effect of nicotine on the proliferation and chondrogenic differentiation of the human Wharton’s jelly mesenchymal stem cells. Biomed Mater Eng.

[22] Kourembanas S (2015). Exosomes: vehicles of intercellular signaling, biomarkers, and vectors of cell therapy. Annu Rev Physiol.

[23] Toh WS, Lai RC, Hui JHP (2017). MSC exosome as a cell-free MSC therapy for cartilage regeneration: implications for osteoarthritis treatment. Semin Cell Dev Biol.

[24] Li K, Yan G, Huang H (2022). Anti-inflammatory and immunomodulatory effects of the extracellular vesicles derived from human umbilical cord mesenchymal stem cells on osteoarthritis via M2 macrophages. J Nanobiotechnol.

[25] Zhang Q, Xiang E, Rao W (2021). Intra-articular injection of human umbilical cord mesenchymal stem cells ameliorates monosodium iodoacetate-induced osteoarthritis in rats by inhibiting cartilage degradation and inflammation. Bone Jt Res.

[26] Ichiseki T, Shimasaki M, Ueda Y (2018). Intraarticularly-injected mesenchymal stem cells stimulate anti-inflammatory molecules and inhibit pain related protein and chondrolytic enzymes in a monoiodoacetate-induced rat arthritis model. Int J Mol Sci.

[27] Peng K, Lu C, Hu S (2020). Therapeutic effect of human umbilical cord mesenchymal stem cells overexpressing miR-140-5p on osteoarthritis. J Shanxi Med Univ.

[28] Zhou H, Shen X, Yan C (2022). Extracellular vesicles derived from human umbilical cord mesenchymal stem cells alleviate osteoarthritis of the knee in mice model by interacting with METTL3 to reduce m6A of NLRP3 in macrophage. Stem Cell Res Ther.

[29] Mianehsaz E, Mirzaei HR, Mahjoubin-Tehran M (2019). Mesenchymal stem cell-derived exosomes: A new therapeutic approach to osteoarthritis?. Stem Cell Res Ther.

[30] Wang Y, Yu D, Liu Z (2017). Exosomes from embryonic mesenchymal stem cells alleviate osteoarthritis through balancing synthesis and degradation of cartilage extracellular matrix. Stem Cell Res Ther.

[31] Morente-Lopez M, Mato-Basalo R, Lucio-Gallego S (2022). Therapy free of cells vs human mesenchymal stem cells from umbilical cord stroma to treat the inflammation in OA. Cell Mol Life Sci.

[32] Mao G, Zhang Z, Hu S (2018). Exosomes derived from miR-92a-3p-overexpressing human mesenchymal stem cells enhance chondrogenesis and suppress cartilage degradation via targeting WNT5A. Stem Cell Res Ther.

[33] Tang S, Chen P, Zhang H (2021). Comparison of curative effect of human umbilical cord-derived mesenchymal stem cells and their small extracellular vesicles in treating osteoarthritis. Int J Nanomed.

[34] Gunay AE, Karaman I, Guney A (2022). Assessment of clinical, biochemical, and radiological outcomes following intra-articular injection of Wharton jelly-derived mesenchymal stromal cells in patients with knee osteoarthritis: a prospective clinical study. Medicine (Baltimore).

[35] Suh DW, Han SB, Yeo WJ (2021). Human umbilical cord-blood-derived mesenchymal stem cell can improve the clinical outcome and Joint space width after high tibial osteotomy. Knee.

[36] Lim HC, Park YB, Ha CW (2021). Allogeneic umbilical cord blood-derived mesenchymal stem cell implantation versus microfracture for large, full-thickness cartilage defects in older patients: a multicenter randomized clinical trial and extended 5-year clinical follow-up. Orthop J Sports Med.

[37] Song JS, Hong KT, Kong CG (2020). High tibial osteotomy with human umbilical cord blood-derived mesenchymal stem cells implantation for knee cartilage regeneration. World J Stem Cells.

[38] Song JS, Hong KT, Kim NM (2020). Human umbilical cord blood-derived mesenchymal stem cell implantation for osteoarthritis of the knee. Arch Orthop Trauma Surg.

[39] Dilogo IH, Canintika AF, Hanitya AL (2020). Umbilical cord-derived mesenchymal stem cells for treating osteoarthritis of the knee: a single-arm, open-label study. Eur J Orthop Surg Traumatol.

[40] Matas J, Orrego M, Amenabar D (2019). Umbilical cord-derived mesenchymal stromal cells (MSCs) for knee osteoarthritis: repeated MSC dosing is superior to a single MSC dose and to hyaluronic acid in a controlled randomized phase I/II trial. Stem Cells Transl Med.

[41] Park YB, Ha CW, Lee CH (2017). Cartilage regeneration in osteoarthritic patients by a composite of allogeneic umbilical cord blood-derived mesenchymal stem cells and hyaluronate hydrogel: results from a clinical trial for safety and proof-of-concept with 7 years of extended follow-up. Stem Cells Transl Med.

[42] Wang Y, Jin W, Liu H (2016). Curative effect of human umbilical cord mesenchymal stem cells by intra-articular injection for degenerative knee osteoarthritis. Zhongguo Xiu Fu Chong Jian Wai Ke Za Zhi.

[43] Yang X, Jiang F, Zhang F, Shi X, Liu L, Xu Z (2017). Controlled study of umbilical cord mesenchymal stem cells in the treatment of severe knee osteoarthritis. Chin Clin Pharmacol Ther.

[44] Chung YW, Yang HY, Kang SJ (2021). Allogeneic umbilical cord blood-derived mesenchymal stem cells combined with high tibial osteotomy: a retrospective study on safety and early results. Int Orthop.

[45] Bartolucci J, Verdugo FJ, Gonzalez PL (2017). Safety and efficacy of the intravenous infusion of umbilical cord mesenchymal stem cells in patients with heart failure: a phase 1/2 randomized controlled trial (RIMECARD trial [randomized clinical trial of intravenous infusion umbilical cord mesenchymal stem cells on cardiopathy]). Circ Res.

[46] Dhillon J, Kraeutler MJ, Belk JW (2022). Umbilical cord-derived stem cells for the treatment of knee osteoarthritis: a systematic review. Orthop J Sports Med.

[47] Gupta A, El-Amin SF, Levy HJ, Sze-Tu R, Ibim SE, Maffulli N (2020). Umbilical cord-derived Wharton’s jelly for regenerative medicine applications. J Orthop Surg Res.

[48] Lo WC, Chen WH, Lin TC (2013). Preferential therapy for osteoarthritis by cord blood MSCs through regulation of chondrogenic cytokines. Biomaterials.

[49] Contentin R, Demoor M, Concari M (2020). Comparison of the chondrogenic potential of mesenchymal stem cells derived from bone marrow and umbilical cord blood intended for cartilage tissue engineering. Stem Cell Rev Rep.

[50] Kim GB, Shon OJ (2020). Current perspectives in stem cell therapies for osteoarthritis of the knee. Yeungnam Univ J Med.

[51] Zhao C, Zhang L, Kong W (2015). Umbilical cord-derived mesenchymal stem cells inhibit Cadherin-11 expression by fibroblast-like synoviocytes in rheumatoid arthritis. J Immunol Res.

[52] Ju Y, Yi L, Li C (2022). Comparison of biological characteristics of human adipose- and umbilical cord- derived mesenchymal stem cells and their effects on delaying the progression of osteoarthritis in a rat model. Acta Histochem.

[53] Ip HL, Nath DK, Sawleh SH (2020). Regenerative medicine for knee osteoarthritis—the efficacy and safety of intra-articular platelet-rich plasma and mesenchymal stem cells injections: a literature review. Cureus.

[54] Gupta A, Maffulli N, Rodriguez HC, Lee CE, Levy HJ, El-Amin SF (2021). Umbilical cord-derived Wharton’s jelly for treatment of knee osteoarthritis: study protocol for a non-randomized, open-label, multi-center trial. J Orthop Surg Res.

